# Brachyury regulates proliferation of cancer cells via a p27^Kip1-^dependent pathway

**DOI:** 10.18632/oncotarget.1999

**Published:** 2014-05-21

**Authors:** Jana Jezkova, Jason S. Williams, Ffion Jones-Hutchins, Stephen J. Sammut, Simon Gollins, Ian Cree, Sarah Coupland, Ramsay J. McFarlane, Jane A. Wakeman

**Affiliations:** ^1^ North West Cancer Research Institute, College of Natural Sciences, Bangor, Gwynedd, UK; ^2^ School of Medical Sciences, Bangor, Gwynedd, UK; ^3^ North Wales Cancer Treatment Centre, Betsi Cadwaladr University Health Board, Bodelwyddan, UK; ^4^ Warwick Medical School, University Hospitals Coventry and Warwickshire, Walsgrave, Coventry, UK; ^5^ Pathology, Department of Molecular and Clinical Cancer Medicine, University of Liverpool, UK; ^6^ NISCHR Cancer Genetics Biomedical Research Unit, Welsh Government, Cathays Park, Cardiff, UK

**Keywords:** Brachyury, proliferation, colorectal cancer, p27Kip1

## Abstract

The T-box transcription factor Brachyury is expressed in a number of tumour types and has been demonstrated to have cancer inducing properties. To date, it has been linked to cancer associated induction of epithelial to mesenchymal transition, tumour metastasis and expression of markers for cancer stem-like cells. Taken together, these findings indicate that Brachyury plays an important role in the progression of cancer, although the mechanism through which it functions is poorly understood. Here we show that Brachyury regulates the potential of Brachyury-positive colorectal cancer cells to proliferate and reduced levels of Brachyury result in inhibition of proliferation, with features consistent with the cells entering a quiescent-like state. This inhibition of proliferation is dependent upon p27^Kip1^ demonstrating that Brachyury acts to modulate cellular proliferative fate in colorectal cancer cells in a p27^Kip1^-dependent manner. Analysis of patient derived colorectal tumours reveals a heterogeneous localisation of Brachyury (in the nucleolus, nucleus and cytoplasm) indicating the potential complexity of the regulatory role of Brachyury in solid colorectal tumours.

## INTRODUCTION

Brachyury (T) is a developmentally regulated T-box transcription factor involved in controlling cell movements and differentiation [1-3]. Brachyury expression has been detected in various cancers including chordoma [4], lung and colon carcinomas [5], where Brachyury protein in the cytoplasm has been linked to high tumour grade and poor prognosis [4, 5]. The role of Brachyury in tumour development is not well understood but it has been shown to induce epithelial to mesenchymal transition (EMT) [6], impose a survival advantage to lung cancer cells [7] and is also linked to the acquisition of properties of cancer stem-like cells (CSCs) [8]. A recent study in chordoma cell lines demonstrated that Brachyury-depletion resulted in G1 growth arrest, through an unknown mechanism, and it was postulated that Brachyury might be a master regulator controlling an oncogenic transcriptional network [9, 10]. Decreased Brachyury has also been shown to lead to decreased cell proliferation, migration and invasion in mice [11].

p27^Kip1^ (encoded by the *CDKN1B* gene) is a member of the Cip/Kip family of cyclin-dependent kinase inhibitors (CKI) which have well-described nuclear-associated tumour suppressor functions in causing G1 cell-cycle phase arrest [12-14]. Evidence also shows a role for p27^Kip1^ in maintaining genomic integrity in the gastrointestinal tissue of mice through control of the transition of G2/M in response to DNA damage by genotoxic agents [15]. Consistent with this, p27^KIP^ is an inhibitor of gastrointestinal tumourigenesis in mice [16] and the tumour suppressor functions associated with p27^Kip1^ may be mediated by inhibition of cell-cycle progression beyond G1 and maintenance of genomic stability in G2/M. In accordance with a function in tumour suppression, loss of p27^Kip1^ in tumour cells is associated with a higher tumour grade and poor prognosis [17-21].

p27^Kip1^ also acts as a multi-functional regulator, and has cyclin-CDK inhibitor-independent functions (linked to its localisation), being involved in alteration of actin dynamics and migration [22-24] and in the control of cell differentiation, acting as a key cell-cycle to differentiation determinant [25-27]. p27^Kip1^ has been shown to be regulated by cMYC at the level of both protein and mRNA [25, 28-31]. cMYC is a major oncogenic driver and has diverse roles in regulation of cell proliferation, growth, apoptosis, metabolism and differentiation [32].

Here, we show that reduction in the levels of Brachyury in colorectal cancer (CRC) cells perturbs proliferation through a mechanism which involves p27^Kip1^ and induces a quiescent-like state from which the cells can recover when grown under suitable growth conditions. Our results place cMYC downstream of Brachyury and suggest that Brachyury modulates the proliferative fate of cells. In studies of patient-derived CRC material a complex relationship between Brachyury and p27^Kip1^ is revealed, based on heterogeneous localisation patterns of Brachyury within the carcinoma. Brachyury is localised to a region of the nucleus, consistent with the nucleolus, and/or the cytoplasm of some, but not all the cells in the carcinoma, suggesting region specific functions within the tumour.

## RESULTS

### *Brachyury* maintains proliferation of CRC cells

**Figure 1:**
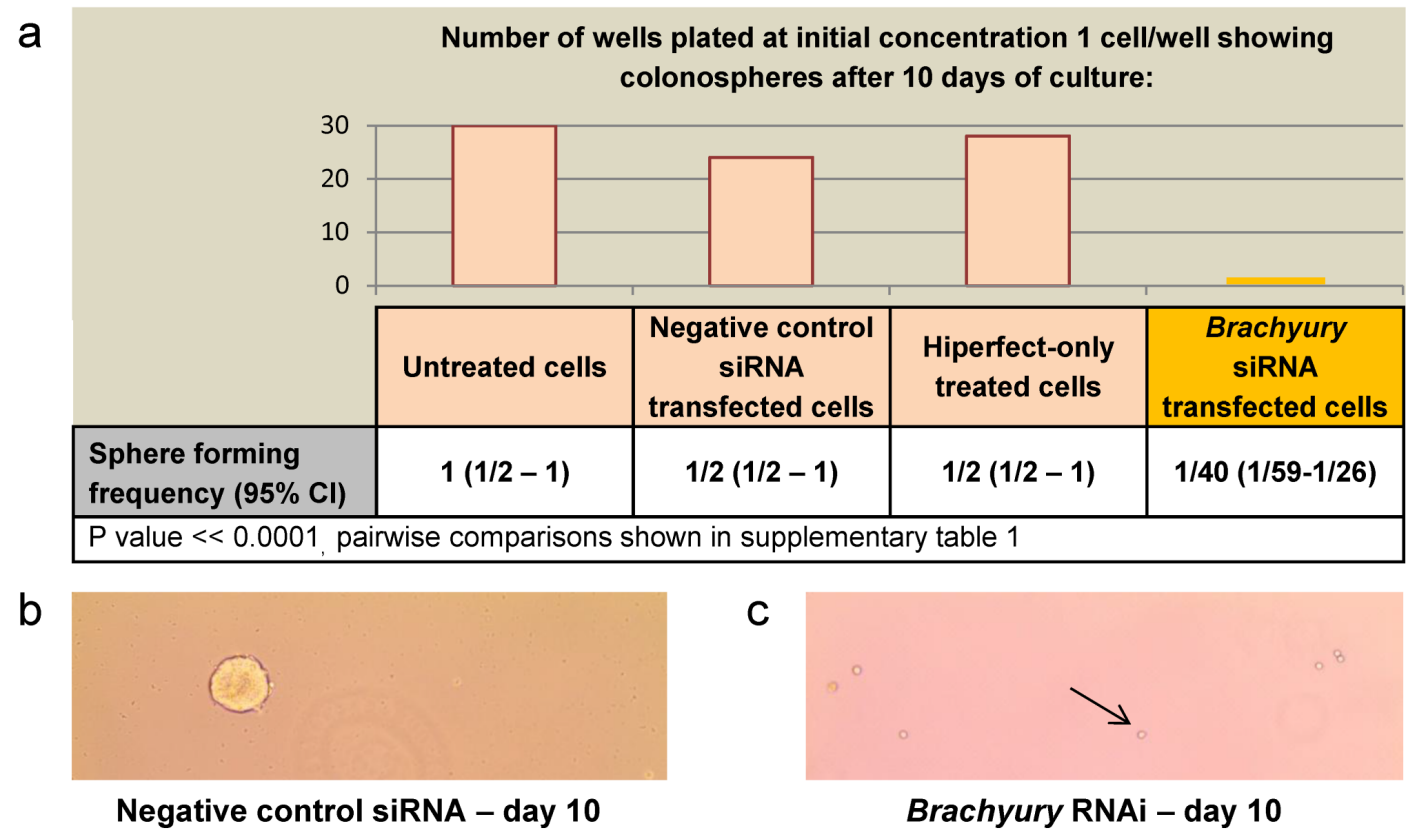
The effect of Brachyury on colorectal cancer cell proliferation as determined by Extreme limiting dilution analysis (ELDA) a) ELDA of colonosphere forming frequency of SW480 CRC cells Colonospheres derived from 1, 10, 100 or 1000 CRC cells (per well) were transfected with negative control, Hs_T_8 or Hs_T_5 *Brachyury* siRNA or hiperfect only, to determine the effect on frequency of sphere formation. Column bars on the graph represent the sphere forming frequencies shown in the table beneath. Brachyury-knockdown levels were routinely around 80% knockdown as assessed by qRT-PCR. CI = confidence interval. Single Brachyury-depleted cells were 20-fold reduced in their sphere forming ability compared to controls. The frequency of sphere forming cells was determined using the ELDA web tool at http://bioinf.wehi.edu.ac/software/elda. The number of wells used and data for each dilution of cells is shown in figure S1. b) Example of spheres formed from single SW480 colorectal cancer cells after 10 days with non-interfering siRNA (control conditions) c) Example of single SW480 colorectal cancer cells that remain in culture after 10 days treatment with siRNA to *Brachyury* (Hs_T_8 or Hs_T_5), the black arrow points to a single cell (x10 magnification).

We were interested to determine whether Brachyury affects proliferation of CRC cells. We used the Brachyury positive CRC cell line, SW480, to derive colonospheres (potentially enriched for cancer progenitor cells), and carried out an extreme limiting dilution assay (ELDA) [34] to determine the ability of single CRC cells to proliferate and form spheres in the presence of Brachyury or under conditions of siRNA-induced Brachyury-depletion. Single Brachyury-depleted CRC cells were 20-fold reduced in their ability to form colonospheres and to proliferate, compared to controls (Figure 1a, S1). In the presence of Brachyury (controls) spheres are formed from single CRC cells (Figure 1b). However, when Brachyury levels are reduced, the single cells that are plated for the proliferation assay remain morphologically unchanged for the duration of the experiment (Figure 1c). The reduced number of spheres formed following *Brachyury-*knockdown is neither due to apoptosis or senescence (S2). In accordance with these observations, the inhibition of proliferation following reduction of Brachyury was reversible, and when *Brachyury*-knockdown single cells were transferred to fresh media they recovered and formed spheres at a frequency of around 30-40%, suggesting that reduced levels of Brachyury result in a recoverable, quiescent-like state in the CRC cells.

### *Brachyury* regulates cell-cycle modulators cMYC and p27^Kip1^

**Figure 2:**
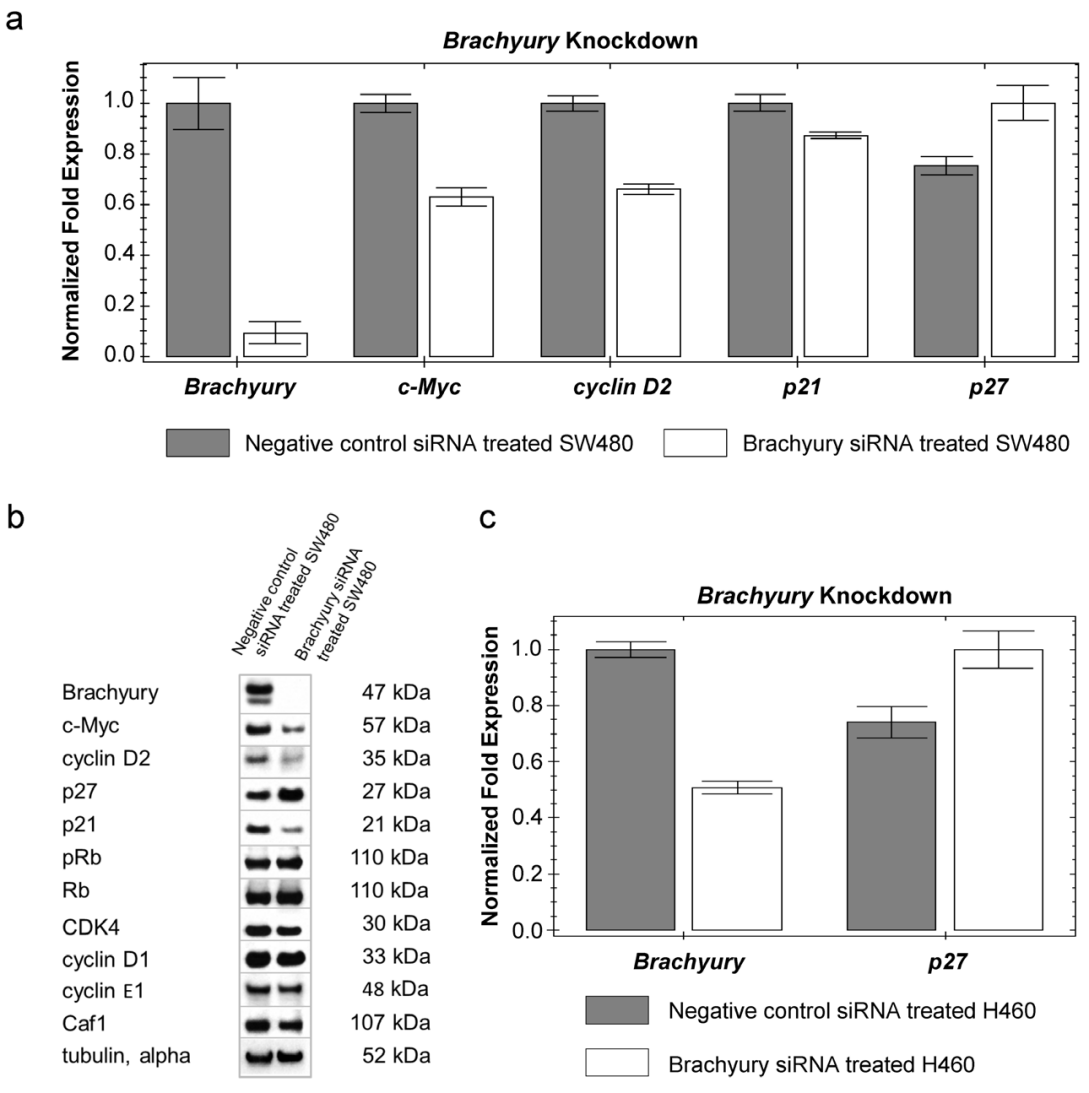
Reduced levels of Brachyury result in increased levels of p27^Kip1^ a) qRT-PCR analysis showing change in expression levels of the panel of genes in response to siRNA knockdown of *Brachyury* in SW480 cells b) Western blot analysis showing changes in levels of proteins in response to siRNA knockdown of *Brachyury* in SW480 cells (Western blot images have been cropped for size and are representative of three repeats). Densitometry measurements for p27^Kip1^ are shown in S3b and reveal a two-fold increase. c) qRT-PCR analysis of *p27*^Kip1^ expression levels in response to siRNA knockdown of *Brachyury* in H460 cells. Results of qRT-PCR analysis were normalised to a combination of three endogenous reference genes (*GAPDH, β-actin* and *lamin A/C*) and the relative fold change in expression was computed by the ΔΔCt method. Error bars show standard error of the mean

We determined whether changes in components of cell-cycle regulatory pathways might be responsible for the cell proliferation-inhibition phenotype we observed following *Brachyury*-knockdown. *Brachyury*-knockdown in CRC cells (grown as spheres) had a profound negative effect on their ability to proliferate (Figure 1a,c); therefore we additionally carried out these studies on cells grown as adherent monolayers which could be grown more easily in larger amounts to obtain sufficient biomaterial to study regulatory mechanisms. Cells grown as spheres or monolayers expressed similar levels of *Brachyury,* indicating that results for CRC cells grown as spheres or monolayers should be comparable (S3a). We observed a consistent down-regulation of cMYC (mRNA and protein) following *Brachyury-*knockdown and a corresponding decrease in levels of *cylcin D2* (*CCND2*), a known target of cMYC [37] (Figure 2a,b). Cyclin D2 complexes with CDK4 to control cell division by hyper-phosphorylating retinoblastoma (Rb) leading to its inactivation [38]. However, we see no change in the levels of either phospho-Rb or unphosphorylated-Rb making it unlikely that the growth inhibitory effects observed in these cells occurs through inactivation of Rb (Figure 2b). Following activation of cMYC, cyclin D2 (in a complex with CDK4) has been shown to sequester p27^Kip1^, thereby limiting its cell-cycle inhibitory activity [37]. On depleting Brachyury we observed an increase in the levels of p27^Kip1^ protein (Figure 2b and S3b) consistent with the decreased levels of cyclin D2 relieving the repression of p27^Kip1^, but also observed an increase in the level of expression of the *CDKN1B* gene suggesting that Brachyury might regulate the levels of p27^Kip1^ at a transcriptional level, independently of sequestration by cyclin D2 (Figure 2a). *Brachyury*-knockdown in the lung carcinoma cell line, H460, showed a similar effect of increasing the levels and expression of p27^Kip1^ (Figure 2c), indicating that the negative regulation of p27^Kip1^ by Brachyury may be a universal mechanism in different *Brachyury*-expressing tumour cell types.

**Figure 3:**
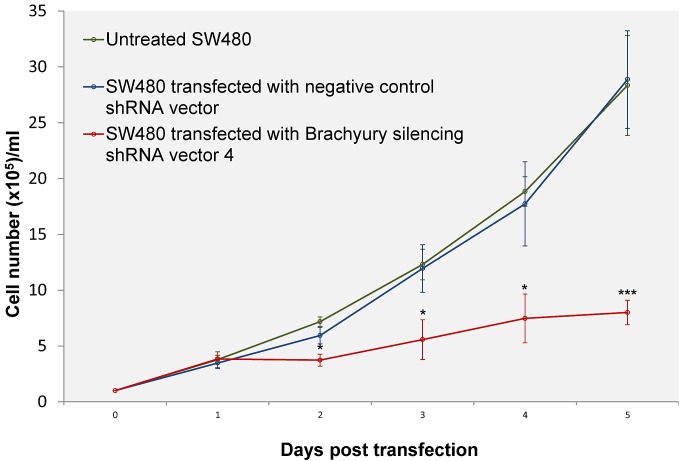
Growth curves of SW480 cells transfected with negative control shRNA vector or silencing shRNA vector (four different shRNA expressing vectors with GFP-selection), compared with untreated SW480 cells Cells transfected with *Brachyury* shRNA expressing vectors revealed elongated cell doubling times (from 24.83± 1.2 hours in control cells to 40.24±2.65 hours in Brachyury depleted cells, mean ± SD, n=3, P=0.001) (see also S4). Error bars denote standard deviation (n=3). The differences between the negative control and *Brachyury-knockdown* were significant at P values *P < 0.05 and ***P < 0.001.

Analysis of SW480 cells transfected with *Brachyury* shRNA expressing vectors revealed elongated mean cell doubling times (Figure 3, S4). The elongated cell-cycle of transfected cells (GFP-gated) was concomitant with increased lengths of S and G2 phases (1.8 times times longer in S phase and 3.4 times longer in G2 compared to controls) (S4).

### Inhibition of proliferation is dependent upon p27^Kip1^

**Figure 4:**
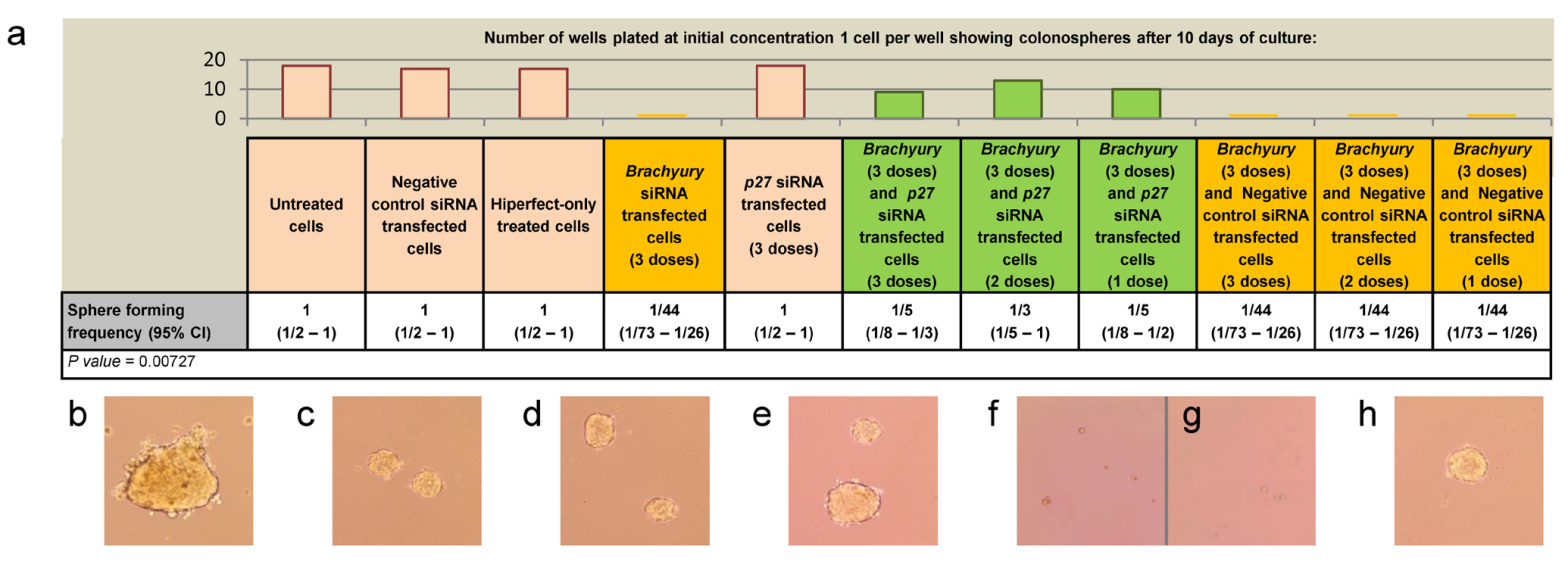
Rescue of Brachyury induced cell proliferation arrest by depletion of *p27*^Kip1^ (a) ELDA assay showing colonosphere forming frequency of colorectal cancer cells under different conditions; control, *Brachyury* single knockdown, *p27*^Kip1^ single knockdown and *Brachyury/p27*^Kip1^ double knockdowns (1, 2 and 3 doses of siRNA). Column bars on the graph represent the sphere forming frequencies shown in the table beneath; *Brachyury*-knockdown cells form spheres at frequencies as low as 1:44 cells. *Brachyury* and *p27^Kip1^* knockdown levels were routinely around 80% as assessed by qRT-PCR. CI = confidence interval. The frequency of sphere forming cells was determined using the ELDA web tool at http://bioinf.wehi.edu.ac/software/elda. The number of wells used and data for each dilution of cells in the ELDA assay are shown in Figure S4. Examples of single SW480 cells grown for 10 days to form spheres are shown in a)-h) under the following conditions b) untreated control cells c) control cells treated with non-interfering siRNA d) control cells treated with hiperfect e) cells treated with siRNA to *p27^Kip1^* f) and g) *Brachyury* siRNA treated cells h) *Brachyury* and *p27^Kip1^* siRNA treated cells

Given the increase in expression levels of p27^Kip1^ following *Brachyury-*knockdown, and the reported ability of p27^Kip1^ to regulate self-renewal of human (h)ES cells [39], we carried out the cell proliferation assay on CRC cells (grown from single cells) under conditions of *Brachyury* and *p27^Kip1^* double knockdown, to determine whether simultaneous p27^Kip1^ reduction could rescue the inhibition of proliferation observed in single *Brachyury-*knockdown cells. Under these conditions, single CRC cells formed spheres close to frequencies of non-interfering control cells (Figure 4a, S5), suggesting that inhibition of p27^Kip1^ may be the predominant pathway through which Brachyury controls CRC cell proliferation. Rescue by p27^Kip1^-reduction could be achieved either by simultaneous knockdown alongside *Brachyury-*knockdown, or following a prior induction of Brachyury proliferation arrest, to induce the quiescent-like state (using siRNA-Brachyury), followed by subsequent knockdown of *p27^Kip1^* at two different time points (Figure 4b-h and S5); both result in proliferative rescue.

**Figure 5:**
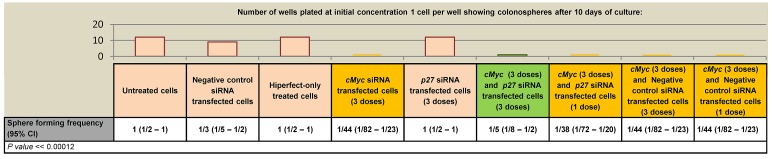
Effect of and siRNA knockdown on SW480 colorectal cancer cell proliferation a) ELDA of colonosphere forming frequency of CRC cells. Colonospheres derived from 1, 10, 100 or 1000 CRC cells/well (SW480) were transfected with negative control non-interfering siRNA, hiperfect only control, *cMyc* siRNA, *p27^Kip1^* siRNA, *cMyc* and *p27^Kip1^*double knockdown siRNA to determine the effect on frequency of sphere formation. Column bars on the graph represent the sphere forming frequencies shown in the table beneath. CI = confidence interval. The frequency of sphere forming cells was determined using the ELDA web tool at http://bioinf.wehi.edu.ac/software/elda b) qRT-PCR analysis of gene expression levels under conditions of cMyc, p27^Kip1^ and cMyc/p27^Kip1^ double knockdown. The number of wells used and data for each dilution of cells is shown in Figure S6.

Decreased levels of cMYC were observed following *Brachyury*-knockdown and so we determined whether similar effects of cell proliferation arrest and induction of a quiescent-like state were imposed following reduction of cMYC or in combination with p27^Kip1^. As before, we carried out a cell proliferation assay on CRC cells, firstly, under conditions of *cMYC*-knockdown. We observed a reduction in the sphere-forming frequency of CRC cells (around a 15 fold reduction in spheres compared to controls spheres) (Figure 5). We then performed *cMYC/p27^KIP^* double knockdown to determine whether p27^Kip1^ could rescue the inhibition of proliferation observed in single *cMYC*-knockdown cells. Whilst simultaneous *cMYC/p27^Kip1^* double knockdown rescues cell proliferation, prior treatment of the cells with *cMYC*-siRNA followed by treatment with *p27^Kip1^*-siRNA could not rescue the negative proliferation effect (Figure 5, S6). In contrast to the results with Brachyury, the cells did not appear to be in a state of quiescence and could not be recovered, suggesting that Brachyury modulates cell proliferation, possibly in a distinct manner from cMYC.

### Brachyury affects expression of cell-cycle regulatory genes

We wanted to further understand the molecular detail of cells with reduced Brachyury and so carried out RNA-seq analysis on control CRC cells versus siRNA *Brachyury-*knockdown cells. There is a pre-dominant down-regulation of genes in *Brachyury-*knockdown cells as revealed by a heat map of the most significantly changed genes organized in hierarchical clusters of gene expression patterns (S7). Some of the most significantly down-regulated genes include those required for progression through the cell-cycle such as *CDC27, CCND2, CCNE1*. Confirming our qRT-PCR data, the *CDKN1B* (p27^Kip1^) gene was up-regulated. Interestingly, a cohort of genes associated with transcription were also down-regulated, such as *TNFRSF12A, POLR3B, POLR2L,* and might reflect the quiescent-like state imposed following *Brachyury-*knockdown which has been associated with decreased RNA levels [40]. Significant changes were observed for gene ontology (GO) clusters (S7: Table 1): these also suggest changes in pathways associated with cell-cycle progression, such as S phase regulators, DNA strand elongation modulators involved in DNA replication, and rRNA processing. Disruption of any of these processes could interfere with the normal cell division cycle and growth, and the reduced levels of RNA may be associated with the induction of quiescence. Associated with this, reduction in RNA levels might also reflect disrupted nucleolar function in *Brachyury-*knockdown cells [41].

### Complex distribution of Brachyury in colorectal carcinomas

**Figure 6:**
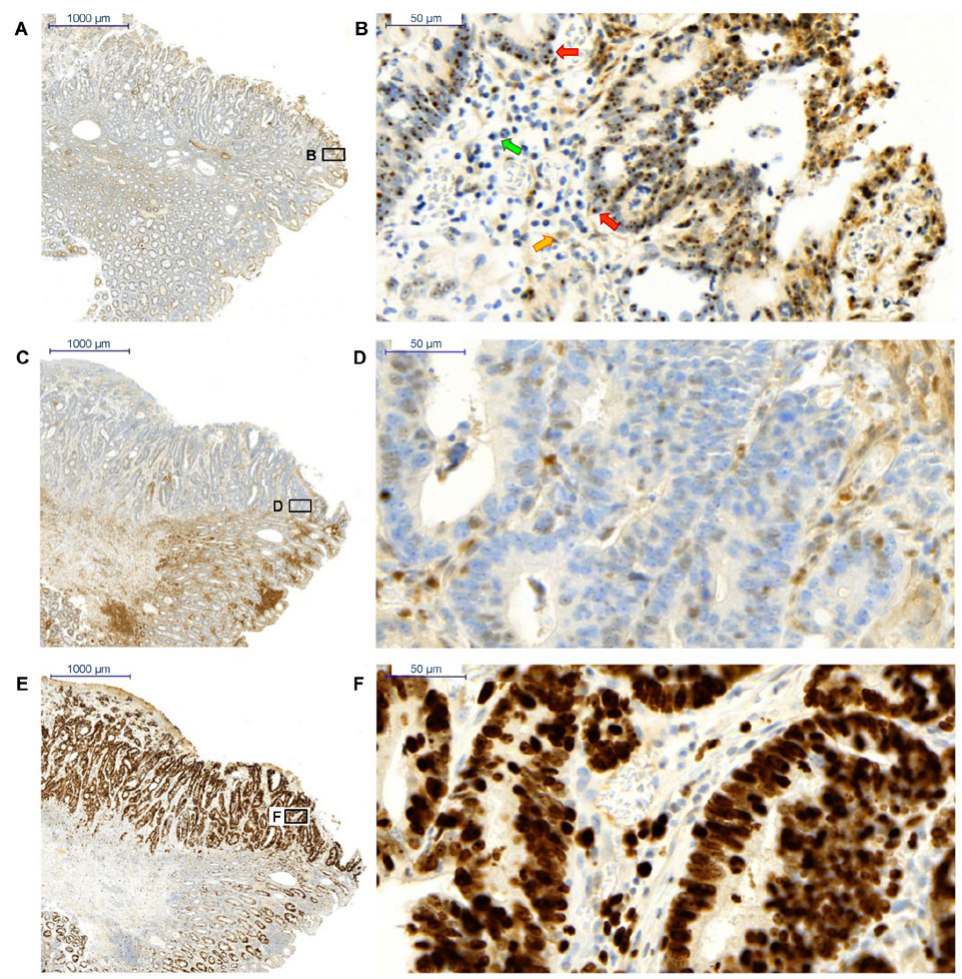
Immunohistochemistry staining of and p27 in a colorectal carcinoma a) and b) *Brachyury* is present in the cytoplasm and nucleolar region of the nucleus in a heterogeneous pattern within the carcinoma (x2 and x40 respectively); red arrows point to nucleolar staining, yellow arrow points to cytoplasmic staining and green arrow points to a negative-staining cell c) and d) p27^Kip1^ is predominantly negative within the carcinoma except for cells at the margins (x2 and x40 respectively) e) and f) Ki67 staining demonstrates proliferation of cells within the carcinoma (x2 and x40 respectively). Four patient samples were studied and all were Brachyury positive: the results shown are from a single patient and are representative of staining patterns obtained for the four Brachyury positive CRC patients.

Our data support the notion that Brachyury modulates the levels of p27^Kip1^ in CRC cells and that this might be important for regulating the proliferative or quiescent potential of cancer cells. Furthermore, GO analysis might infer a role for Brachyury consistent with RNA processing and cell-cycle control. We therefore wanted to compare distribution patterns of Brachyury and p27^Kip1^ in patient-derived CRC specimens by immunohistochemistry (IHC). Not all cells within the carcinoma give positive staining for Brachyury, but some areas displayed distinct nucleolar staining (Figure 6a, b). To our knowledge, this is the first report of this staining pattern for Brachyury. We also observed widespread cytoplasmic staining of Brachyury in accordance with observations by Kilic and co-workers [4]. Whilst the carcinoma was also strongly positive for the proliferation marker Ki67, it only displayed p27^Kip1^ staining at the tumour margin (Figure 6c-f). In summary, the carcinoma revealed heterogeneity of Brachyury localization, stained positive for Ki67, but p27^Kip1^ staining was only observed at the margin. The heterogeneity of Brachyury distribution in the tumour suggests that it may have region specific functions.

## DISCUSSION

We report that Brachyury modulates cancer cell proliferation through a p27^Kip1^-dependent mechanism. When Brachyury levels are reduced, proliferative arrest ensues and a quiescent-like state is adopted from which cells can reversibly recover under suitable growth conditions. This suggests that inhibition of p27^KIP^ is a major route through which Brachyury functions to allow proliferation, since double knockdown of *Brachyury* and *p27^Kip1^* results in restoration of cell proliferation. The effects of Brachyury on p27^Kip1^ might be due to cMYC; whilst *cMYC*-knockdown induces proliferation arrest, there is a difference in the response to Brachyury since cells do not enter a quiescent-like state (as defined by ability to re-enter the cell-cycle). Simultaneous reductions in p27^Kip1^ and cMYC rescue the proliferation defect, however, the arrest imposed by reduced cMYC can only be relieved by simultaneous knockdown of *p27^Kip1^* and not if p27^Kip1^ is depleted after prior growth arrest by *cMYC*-knockdown. The difference in responses between *Brachyury/p27^Kip1^* and *cMYC/p27^Kip1^* double knockdowns may be due to dosage of cMYC. Although reduced, levels of *cMYC* remain relatively high following *Brachyury-*knockdown compared to levels of *cMYC* following *cMYC*-knockdown directly. We might speculate that very low levels of *cMYC* (observed in direct *cMYC*-knockdown) exclude the cells from re-entering the cell-cycle, but higher levels observed in the *Brachyury-*knockdown cells are ‘just right’ to allow cells to enter a quiescent-like state. Evidence in support of this is seen by the fact that one of the main routes by which cMYC drives tumourigenesis is through inhibition of differentiation and it has been shown to antagonize differentiation and growth inhibition effects of p27^Kip1^ in leukaemia cells [42].

**Figure 7:**
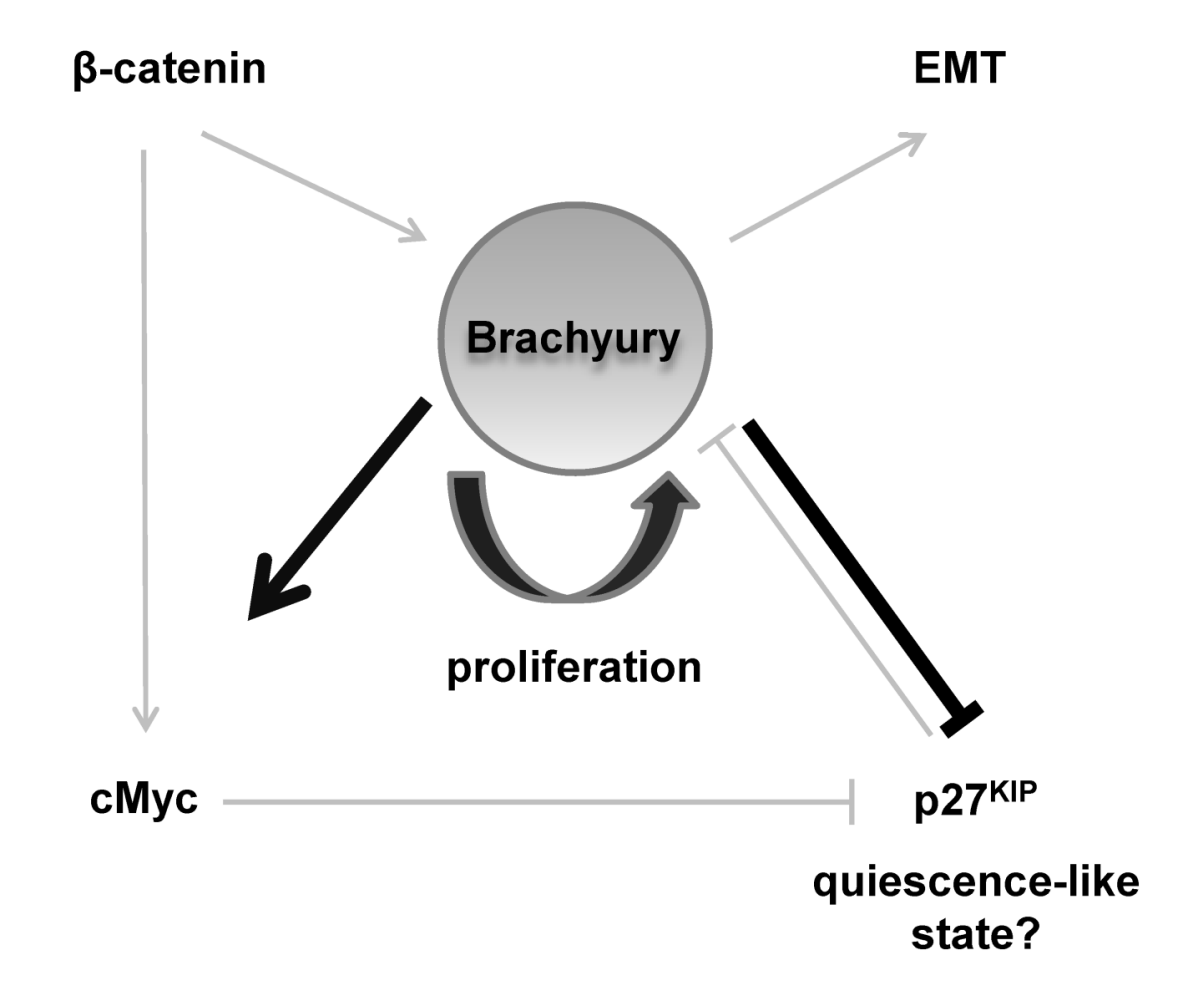
Schematic model describing a central role for Brachyury in regulating proliferation and quiescence

It is possible that Brachyury acts to regulate both. We speculate that the presence of Brachyury results in high cMYC/low p27^Kip1^ allowing proliferation (and avoidance of differentiation), but when Brachyury levels are reduced, this results in low, or optimal levels of, cMYC/high p27^Kip1^ inducing a quiescent-like state, and enabling subsequent cell differentiation with appropriate signals. Brachyury might impose a state of ‘competence’ to cells, allowing decisions to be made between proliferation and quiescence, regulating levels of p27^Kip1^ as a key effector (Figure 7). Interestingly, ChIP-seq experiments performed in mouse identified Brachyury binding sites in Myc and CDKN1B (p27^Kip1^) at proximal enhancer elements [43].

Over-expression of p27^Kip1^ in hES cells leads to growth arrest and a decrease in *Brachyury*, which may be linked to regulation of EMT [39]. Increased p27^Kip1^ is also required to maintain terminal differentiation following exit from the cell-cycle [44]. Our studies show that *Brachyury*-knockdown results in inhibition of proliferation associated with an increased cell doubling time, but do not reveal a specific cell-cycle stage arrest. Studies in chordoma cells, with reduced Brachyury, showed a specific proliferation and G1 phase arrest [10]. The elongation/delayed cell division times that we observed following *Brachyury*-knockdown, and our GO analysis suggests that Brachyury might also be linked to cell growth changes in CRC cells. Interestingly, neither the response to reduced Brachyury in chordomas, nor the response we observe in CRC cells complies with studies carried out recently in lung cell lines where elevated Brachyury was associated with decreased cell proliferation [7]. This may reflect differences inherent to the use of different tumour cell types, or to variation in control of cell-cycle elements by Brachyury in different cell contexts and possibly to different doses, levels or compartmentalization of Brachyury.

High levels of p27^Kip1^ are found in quiescent cells and transcripts are rapidly degraded following mitogenic activation in cancer cells [45]. Indeed, levels of p27^Kip1^ are shown to be important for regulating entry into quiescence [29] and although levels remain high in quiescent cells, there is evidence to suggest that the quiescent state (in fibroblasts at least) is only attained by active suppression of terminal differentiation [46]. Quiescent cells are thought to be ‘poised’, ready to be activated [40] and it could be envisaged that this state is vital for progression of a tumour to a new site. It remains a question as to whether Brachyury is a pre-requisite for cancer cells to modify themselves prior to imposition of a quiescent-like state. Certainly our GO analysis are indicative of changes in genes associated with cell-cycle progression, DNA replication and RNA processing, in agreement with changes that might be observed in the quiescent stem cell signature [40]. Furthermore, the intriguing localization of Brachyury to the nucleolus suggests a role in rRNA processing and/or cell-cycle regulation.

The sub-cellular localization pattern of Brachyury within the carcinoma was complex, possibly reflecting a dynamic contribution to diverse functions in the cell, indeed, Brachyury was recently suggested to act as a master regulator in chordoma cells [10].

We note that not all cancer cells require Brachyury for proliferation, and, Brachyury may just confer ‘competence’ to cells to make ‘decisions’ of whether to proliferate or leave the cell-cycle and differentiate. We also carried out the ELDA assay with the Brachyury-negative CRC cell line, HCT116, and did not observe reduced sphere forming frequencies with *Brachyury-*knockdown. This suggests that alternative mechanisms governing proliferation exist in different CRC cells. Interestingly, HCT116 cells appeared to be nullipotent in differentiation assays of CSCs [47] suggesting again, that the presence of Brachyury might be required to regulate the interface between proliferation and differentiation. In accordance, evidence also suggests that Brachyury is critical in controlling both cell differentiation and division during normal development of the notochord in ascidians [48].

Finally, we postulate that ‘fine tuning’ of Brachyury levels in a cell could be a mechanism for cancer cells to become resistant to chemotherapies: cells would be able to ‘drop out’ of proliferation and enter a quiescent-like state thereby avoiding genotoxic agents targeted at dividing cells. Then, when conditions allow, Brachyury would be ‘switched’ back on, conferring this same state of ‘competence’ and enabling its cancer promoting properties.

## MATERIALS AND METHODS

### Western Blot

These were carried out as in S9. Primary antibodies are listed in S9.

### Quantitative Real-time PCR

RNA was extracted using RNeasy Plus Mini Kit (Qiagen, #74136). First-strand cDNA synthesis used Quantitect Reverse Transcription kit (Qiagen, #205310). qRTPCR was carried out using CFX96 Real-Time System C1000 Thermal Cycler (BioRad) with Quantifast SYBR green RT-PCR kit (Qiagen, #204154). The QuantiTect Primer Assay (Qiagen) was used for genes listed in S9.

### Cell Culture

SW480 cells (ECACC, cell line authentication report number 710236782) were cultured in DMEM medium (Life Technologies, #61965) supplemented with 10% fetal calf serum; H460 cells (ECACC, cell line authentication report number 710236782) were grown in RPMI medium (Life Technologies, #61870) supplemented with 10% fetal calf serum. Both cell lines have undergone 16 loci STR authentication (LGC Standards, UK).

### Transfection

Brachyury siRNA (Qiagen, SI04133521, SI00738255), p27Kip1 (Qiagen, SI02621990), cMYC (Qiagen, SI00300902) and negative control siRNA (Qiagen, 1027280) was used at a final concentration of 5 nM. Transfection was carried out with HiPerFect Reagent (Qiagen, 301705) according to the manufacturer's instructions.

### Colonosphere Formation Assay

Primary colonospheres were generated as previously described [33]. After 5-7 days the colonospheres were dissociated to a single cell suspension with Stem Pro Accutase (Life Technologies, A11105-01) and sub-cultured or used for further experiments.

### Extreme limiting dilution analysis

Extreme limiting dilution analysis (ELDA) was performed as described [34] and details are listed in S9.

### FACS

SW480 cells were transiently transfected with GFP-encoding Sure Silencing shRNA plasmid (Qiagen, KH02753) using Attractene Transfection Reagent (Qiagen, 301005). Target sequences for shRNA and FACS analysis is described in S9.

### Growth curve analysis

SW480 cells were plated at equivalent densities (1 × 10^5^ cells/ml) in 6-well plates and allowed to attach. Transient transfection with shRNA was performed on the same day. A second shRNA treatment and media change was performed day 3 post-transfection. At daily intervals, cells were trypsinized, and 3 wells per condition were counted using Biorad's TC 20 automated cell counter with the trypan blue exclusion method.

### Immunohistochemistry (IHC)

Samples of human colon cancer were obtained from individuals undergoing colonic resection. IHC details are described in S9. Antigen retrieval and antibody dilutions are listed in S9.

### RNAseq

RNA isolation, quality control and details of RNAseq are described in S9. Fastq data underwent guided alignment to the human genome (NCBI Build 37.2) using Tophat v2.0.6 [35]. Read duplicates were removed using Picard (http://picard.sourceforge.net/) and counts/gene generated using HTSeq. Differential expression at the gene level was carried out using the Bioconductor package DESeq [36]. Pathway analysis was carried out on significantly differentially expressed genes using TopGO package (Bioconductor, http://www.bioconductor.org/packages/2.12/bioc/html/topGO.html).

## SUPPLEMENTARY FIGURES AND TABLES


