# Supplementary material for: Brachyury regulates proliferation of cancer cells via a p27Kip1-dependent pathway
Source: Oncotarget. 2014 May 21;5(11):3813–22. doi: 10.18632/oncotarget.1999 (PMC4116522; doi:10.18632/oncotarget.1999)
Supplement: Supplementary file 1 [file oncotarget-05-3813-s001.pdf]

Brachyury regulates proliferation of cancer cells via a p27Kip1-dependent pathway Short title: Brachyury and proliferation

Number of wells plated at initial concentration 1 cell per well showing colonospheres after 10 days of culture:

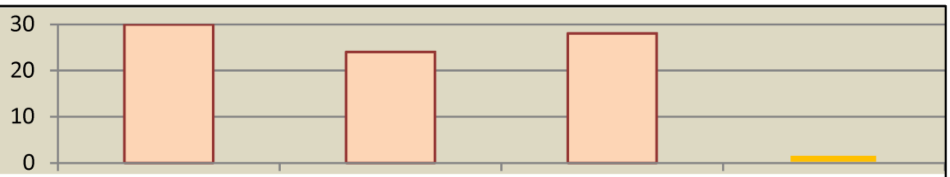

Number of cells per well	Number of wells plated	Untreated cells	Negative control siRNA transfected cells	Hiperfect-only treated cells	*Brachyury* siRNA transfected cells
		Number of wells showing colonospheres after 10 days of culture			
1000	30	30	30	30	30
100	30	30	30	30	30
10	30	30	30	30	2
1	30	30	24	28	0
**Sphere forming frequency (95% CI)**		**1 (1/2 – 1)**	**1/2 (1/2 – 1)**	**1/2 (1/2 – 1)**	**1/40 (1/59-1/26)**
P value	<< 0.0001, pairwise comparisons shown below				

Pairwise tests for differences in cancer cell self-renewal frequencies:

Group 1	Group 2	Chisq	DF	Pr(>Chisq)
B	H	178	1	<< 0.0001
B	N	147	1	<< 0.0001
B	U	200	1	<< 0.0001
H	N	2.4	1	0.121
H	U	2.84	1	0.0919
N	U	8.99	1	0.00272

Pairwise comparison of treatment groups of SW480 cells for differences in self-renewal frequencies.
B - *Brachyury* siRNA transfected cells, U - Untreated cells, N - Negative control siRNA transfected cells,
H - Hiperfect-only treated cells.
Knockdown was carried out on colonospheres grown to different passages (P20.1, P44.7, P44.12 & P44.15).
The table displays the Chi-Square statistic, degrees of freedom (DF), and the probability value (Pr > ChiSq),
with a value below 0.05 indicating a significant difference.

Supplementary fig1: ELDA assay showing the effect of Brachyury siRNA-mediated knockdown in SW480 CRC cells

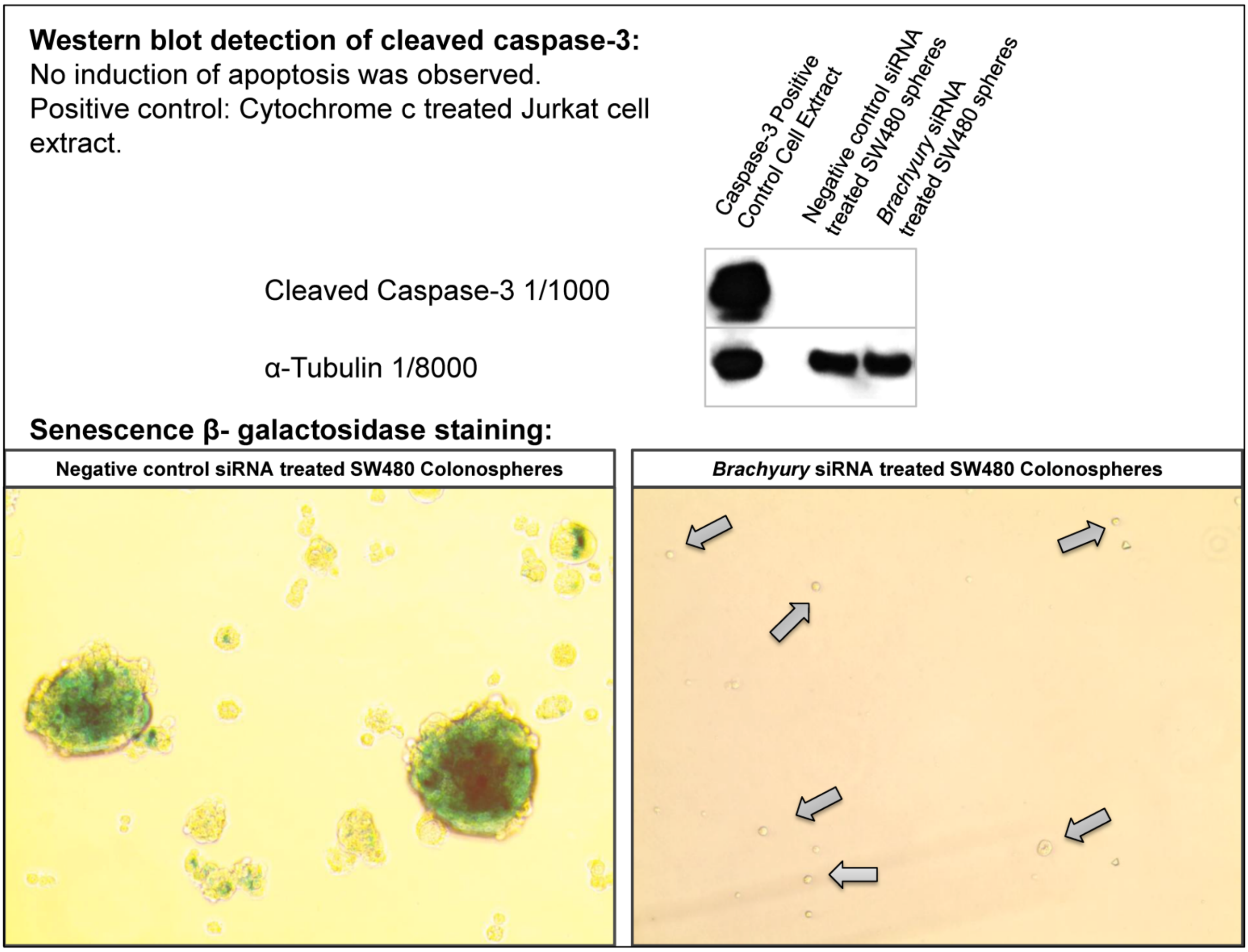

Supplementary fig2: Brachyury-depletion in CRC cells does not result in apoptosis or senescence

Western blot analysis of cleaved caspase 3 shows no induction of apoptosis. This is compliant with our observation that sinlge cells depleted of Brachyury remain morphologically unchanged for the duration of the experiment.

Senescence staining was performed using a Senescence β- galactosidase staining kit (Cell Signaling, #9860) following the manufacturer's protocol. The Brachyury-depleted cells do not undergo senescence as assessed by this assay. This is compliant with our observation that the cells recover following removal of siRNA-Brachyury.

Brachyury knockdown:

Single cell suspension of colonosphere-derived SW480 cells was plated onto 10-cm-diameter ultra-low culture dishes (Costar, 3474) at a concentration of 1×10^5 cells in 10 ml of serum-free stem cell medium. Brachyury siRNA (Qiagen, SI04133521) and negative control siRNA (Qiagen, 1027280) was used at a final concentration of 5 nM. Transfection was carried out with HiPerFect Reagent (Qiagen, 301705) according to the manufacturer’s instructions and transfection complexes were re-applied after 4 and 8 days of incubation. Cells were stained on day 10 of incubation. Media was changed on days 4 and 8 of incubation.

a. SW480 CRC cells grown as monolayers or spheres express similar levels of Brachyury as shown by qRT-PCR

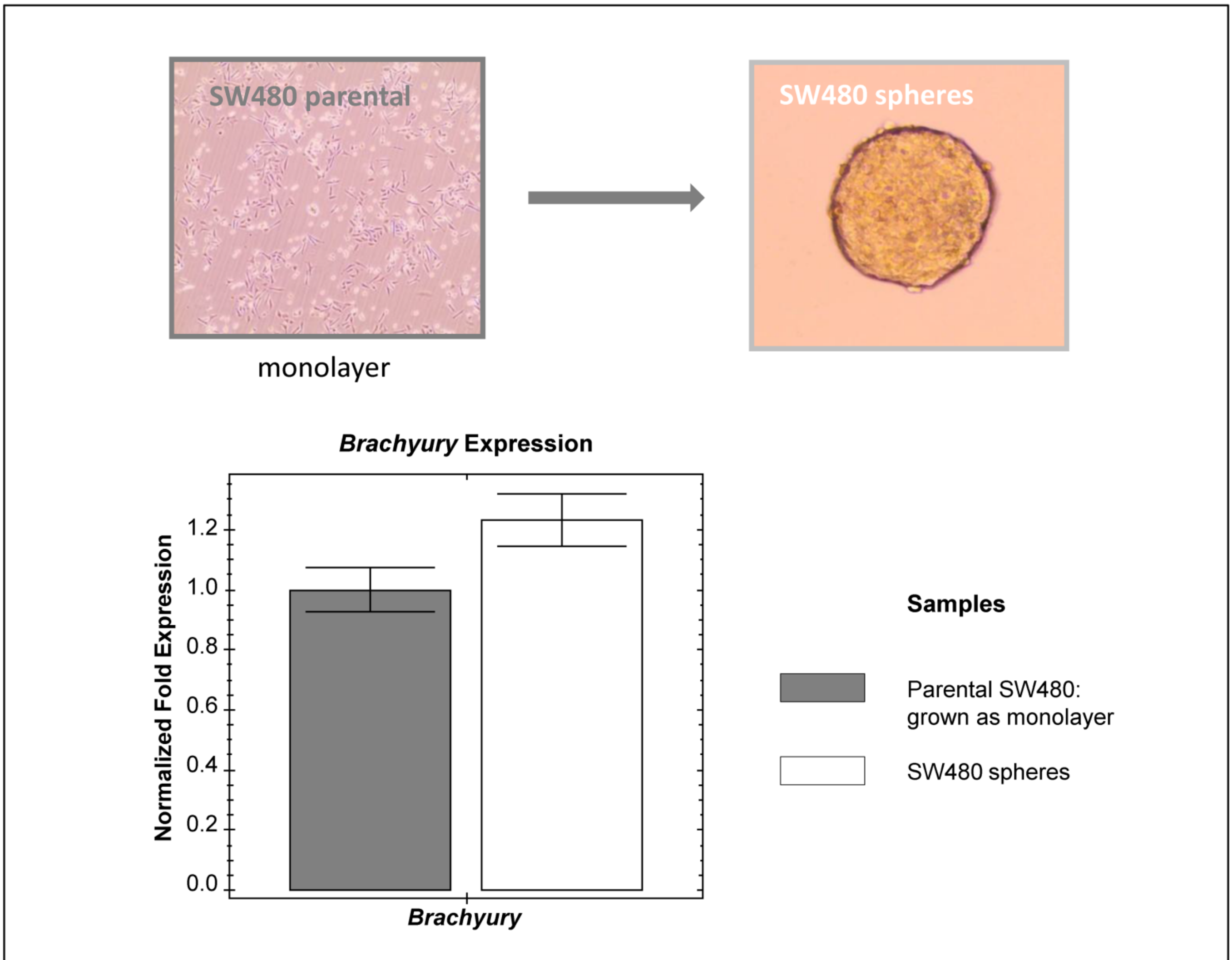

Results were normalised to a combination of three endogenous reference genes (*GAPDH, β-actin* and *lamin A/C*) and the relative fold change in expression was computed by the ΔΔCt method. Error bars show standard error of the mean.

b.

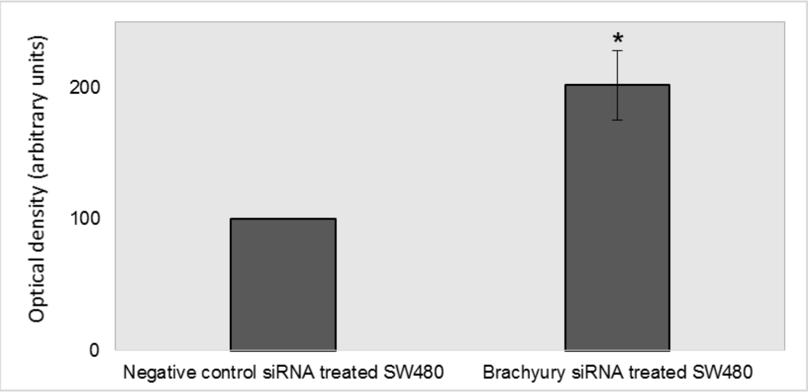

Supplementary fig3: Densitometric quantification of p27^{Kip1} western blots showing a 2.02 mean relative increase in density in Brachyury-depleted conditions compared to controls (n= 3, mean = 202, ±SEM=26.4). *P=0.018 vs control. Analysis was performed using background-subtracted densitometric tools of the Image Lab software (BioRad).

Growth curve analysis - viable-cell counts

Untreated SW480

Days post transfection	0	1	2	3	4	5	Doubling time (hrs)
Cell number (x10⁵)/ml	1.00	3.01	7.67	13.80	19.70	25.60	25.65
	1.00	3.94	6.90	12.00	17.30	25.90	25.56
	1.00	4.38	6.96	11.10	19.50	33.50	23.69
**Mean number of cells (x10⁵)/ml**	**1.00**	**3.78**	**7.18**	**12.30**	**18.83**	**28.33**	**24.97**
Standard deviation	0.00	0.70	0.43	1.37	1.33	4.48	1.11
Mean viability %	100	100	97	100	92	92	

SW480 transfected with negative control shRNA vector

Days post transfection	0	1	2	3	4	5	Doubling time (hrs)
Cell number (x10⁵)/ml	1.00	3.34	5.20	12.70	20.20	30.60	24.31
	1.00	4.00	5.92	13.60	19.60	32.10	23.98
	1.00	3.07	6.68	9.53	13.40	23.90	26.21
**Mean number of cells (x10⁵)/ml**	**1.00**	**3.47**	**5.93**	**11.94**	**17.73**	**28.87**	**24.83**
Standard deviation	0.00	0.48	0.74	2.14	3.76	4.37	1.20
Mean viability %	100	99	98	99	99	98	

SW480 transfected with *Brachyury* silencing shRNA vector 4

Days post transfection	0	1	2	3	4	5	Doubling time (hrs)
Cell number (x10⁵)/ml	1.00	3.62	3.51	3.78	9.48	6.96	42.87
	1.00	3.67	3.34	7.34	5.15	7.89	40.27
	1.00	4.22	4.34	5.59	7.78	9.15	37.57
**Mean number of cells (x10⁵)/ml**	**1.00**	**3.84**	**3.73**	**5.57**	**7.47**	**8.00**	**40.24*** **
Standard deviation	0.00	0.33	0.54	1.78	2.18	1.10	2.65
Mean viability %	100	94	91	93	94	97	

***P = 0.001 compared with controls.

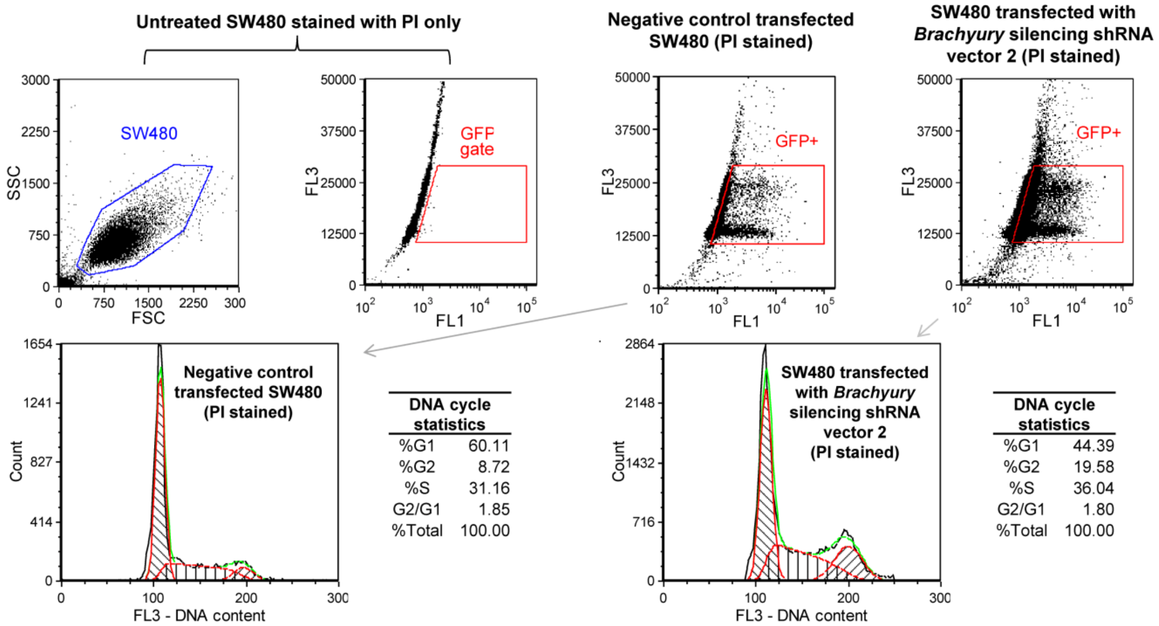

	Live cell count	Viability	Doubling time (hrs)
**Untreated SW480**	9.48x10⁵	98%	22.19
**Negative control shRNA**	1.02x10⁶	100%	21.49

Brachyury knockdown	Live cell count	Viability	Doubling time (hrs)
**shRNA 1**	4.05x10⁵	96%	35.68
**shRNA 2**	4.66x10⁵	97%	32.43
**shRNA 3**	3.07x10⁵	90%	44.49
**shRNA 4**	3.34x10⁵	95%	41.38

Supplementary fig4: SW480 cells were transfected either with a negative control + GFP or with *Brachyury* silencing + GFP shRNA vector. For the flow-cytometric analysis GFP-positive cells were gated and cell cycle profiles were analysed with the multicycle cell cycle analysis plug-in for FCS Express 4. Four different *Brachyury* silencing shRNAs were used (shRNA vector 1-4), FACS profiles for negative control vector and shRNA vector 2 are shown as representative plots for three separate experiments.

a

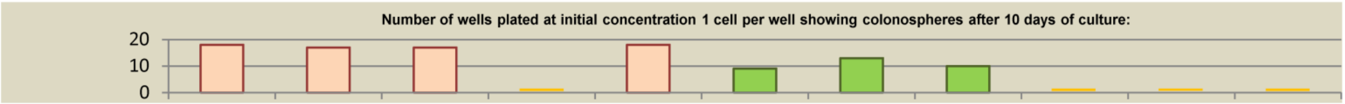

Number of cells per well	Number of wells plated	Untreated cells	Negative control siRNA transfected cells	Hiperfect-only treated cells	*Brachyury* siRNA transfected cells (3 doses)	*p27* siRNA transfected cells (3 doses)	*Brachyury* (3 doses) and *p27* siRNA transfected cells (3 doses)	*Brachyury* (3 doses) and *p27* siRNA transfected cells (2 doses)	*Brachyury* (3 doses) and *p27* siRNA transfected cells (1 dose)	*Brachyury* (3 doses) and Negative control siRNA transfected cells (3 doses)	*Brachyury* (3 doses) and Negative control siRNA transfected cells (2 doses)	*Brachyury* (3 doses) and Negative control siRNA transfected cells (1 dose)
		Number of wells showing colonospheres										
1000	18	18	18	18	18	18	18	18	18	18	18	18
100	18	18	18	18	18	18	18	18	18	18	18	18
10	18	18	18	18	0	18	14	16	14	0	0	0
1	18	18	17	17	0	18	9	13	10	0	0	0
Sphere forming frequency (95% CI)		1 (1/2 – 1)	1 (1/2 – 1)	1 (1/2 – 1)	1/44 (1/73 – 1/26)	1 (1/2 – 1)	1/5 (1/8 – 1/3)	1/3 (1/5 – 1)	1/5 (1/8 – 1/2)	1/44 (1/73 – 1/26)	1/44 (1/73 – 1/26)	1/44 (1/73 – 1/26)
P value	0.00727											

b

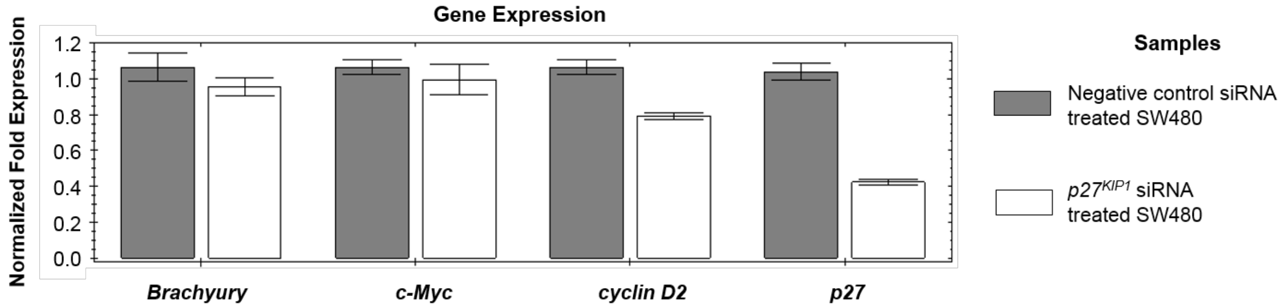

c

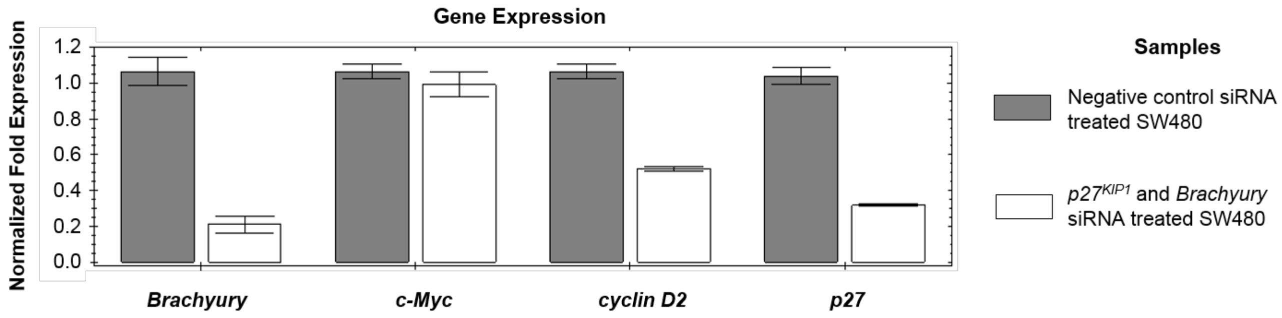

Supplementary fig5: qRT-PCR results were normalised to a combination of three endogenous reference genes (*GAPDH, β-actin* and *lamin A/C*) and the relative fold change in expression was computed by the ΔΔCt method. Error bars show standard error of the mean.

S 6.

a

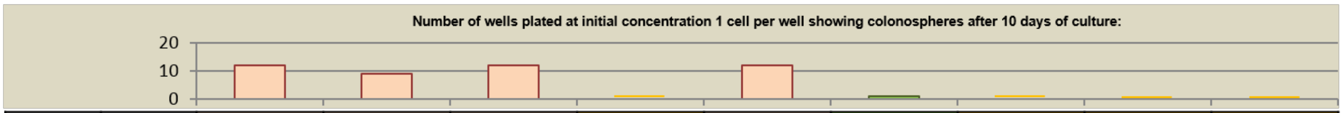

Number of cells per well	Number of wells plated	Untreated cells	Negative control siRNA transfected cells	Hiperfect-only treated cells	*cMyc* siRNA transfected cells (3 doses)	*p27* siRNA transfected cells (3 doses)	*cMyc* (3 doses) and *p27* siRNA transfected cells (3 doses)	*cMyc* (3 doses) and *p27* siRNA transfected cells (1 dose)	*cMyc* (3 doses) and Negative control siRNA transfected cells (3 doses)	*cMyc* (3 doses) and Negative control siRNA transfected cells (1 dose)
		Number of wells showing colonospheres								
1000	12	12	12	12	12	12	12	12	12	12
100	12	12	12	12	12	12	12	12	12	12
10	12	12	11	12	0	12	12	1	0	0
1	12	12	9	12	0	12	1	0	0	0
Sphere forming frequency (95% CI)		1 (1/2 – 1)	1/3 (1/5 – 1/2)	1 (1/2 – 1)	1/44 (1/82 – 1/23)	1 (1/2 – 1)	1/5 (1/8 – 1/2)	1/38 (1/72 – 1/20)	1/44 (1/82 – 1/23)	1/44 (1/82 – 1/23)
P value	<< 0.00012									

b

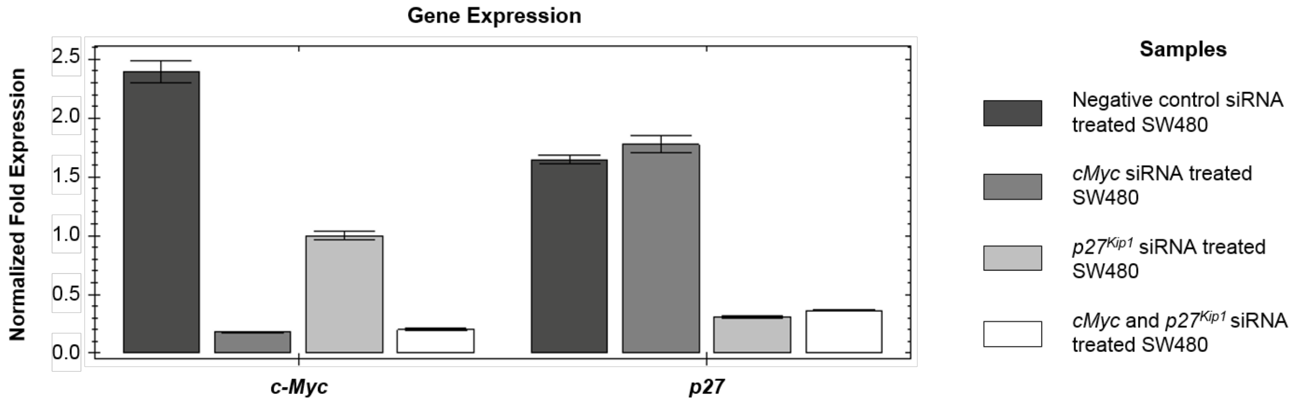

Supplementary fig6: qRT-PCR results were normalised to a combination of three endogenous reference genes (*GAPDH, β-actin* and *lamin A/C*) and the relative fold change in expression was computed by the ΔΔCt method. Error bars show standard error of the mean.

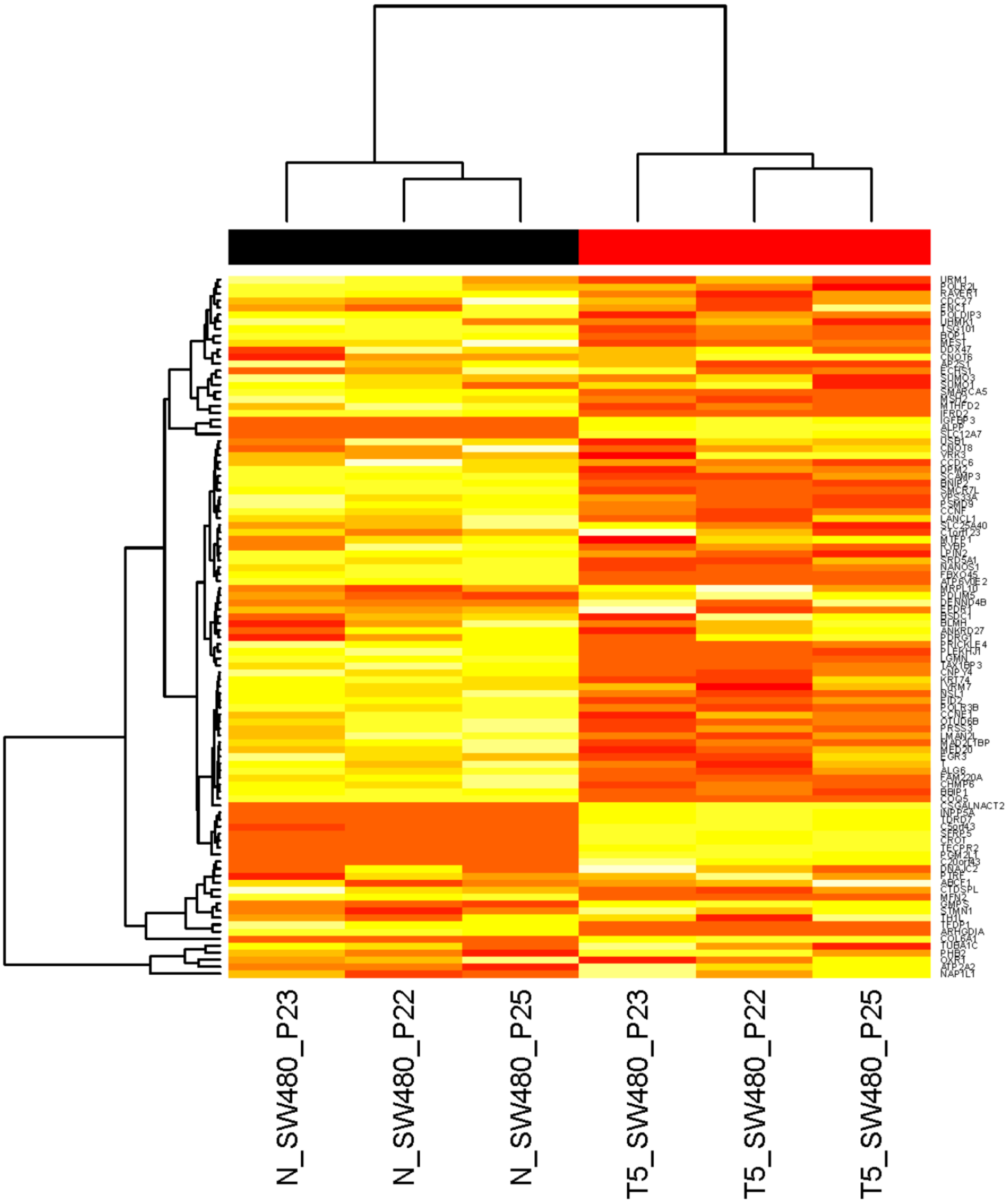

N – SW480 transfected with negative control siRNA, T – siRNA Brachyury knockdown).
Red color shows low expression level, while yellow marks high expression level.

Supplementary fig7: Heat map demonstrating the top 100 most differentially expressed genes in the biological replicates of N and T conditions. RNA-seq conditions are described in S9.

Supplementary Table 1: Enriched molecular function categories in GO analysis

GO ID	Term	Annotated	Significant	Expected	P-value classic
GO:0000084	S phase of mitotic cell cycle	141	49	31.84	0.00125
GO:0006271	DNA strand elongation involved in DNA replication	31	18	7	4.20E-05
GO:0006364	rRNA processing	105	37	23.71	0.00038
GO:0006400	tRNA modification	27	14	6.1	0.00169
GO:0007050	cell cycle arrest	371	113	83.78	0.00194
GO:0007162	negative regulation of cell adhesion	84	33	18.97	0.00164
GO:0009070	serine family amino acid biosynthetic process	13	7	2.94	0.00106
GO:0010811	positive regulation of cell-substrate adhesion	57	21	12.87	0.00095
GO:0030198	extracellular matrix organization	232	81	52.39	0.00026
GO:0032508	DNA duplex unwinding	27	13	6.1	0.00099
GO:0032535	regulation of cellular component size	164	55	37.04	0.00209
GO:0035019	somatic stem cell maintenance	37	12	8.36	0.00132
GO:0045892	negative regulation of transcription DNA-dependent	753	199	170.05	0.0018
GO:0048008	platelet-derived growth factor receptor signaling pathway	35	18	7.9	0.00207
GO:0048844	artery morphogenesis	42	14	9.48	0.00092
GO:0050852	T cell receptor signaling pathway	81	18	18.29	0.00094
GO:0051028	mRNA transport	102	33	23.03	0.00071
GO:0060491	regulation of cell projection assembly	52	20	11.74	0.00174
GO:0061077	chaperone-mediated protein folding	23	7	5.19	0.00128

S 8. Antibody validation (siRNA depletion of Brachyury and western blot)

Western blot: These antibodies were used in western blots during this study:

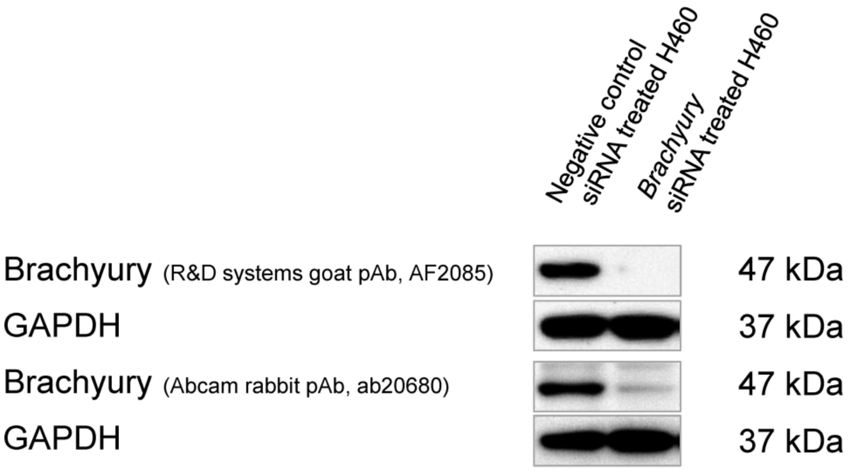

Immunohistochemistry: List shows antibodies used for IHC in this study and other studies:

Primary Antibody	Cat. no.	Source	Host	Clonality	Reference
Anti-Brachyury	ab57480	Abcam	Mouse	Monoclonal	Roselli et al., 2012 (IHC, RNAi-shRNA); Rinner et al., 2012 (IHC)
Anti-Brachyury	ab20680	Abcam	Rabbit	Polyclonal	Shen et al., 2011 (IHC); Haro et al., 2013 (IHC)
Anti-Brachyury	AF2085	R&D systems	Goat	Polyclonal	Kilic et al., 2011 (IHC)

S9.

Western Blot Whole-cell lysates were prepared using M-PER lysis buffer (Thermo Scientific, #78503), Halt Protease Inhibitor Cocktail (Thermo Scientific, #87785) and Halt Phosphatase Inhibitor Cocktail (Thermo Scientific, #78420). Fast Western Blot Kit, ECL substrate (Thermo Scientific, #35050) was used according to manufacturer's instructions to detect the rabbit and mouse primary antibodies (listed in Supplementary Table 4). Goat polyclonal antibodies**:** membranes were probed with primary antibodies in 10% dry milk/PBS/0.5% Tween 20. Incubation with secondary antibodies was performed at room temperature for 1 hour, followed by a 10 minute wash in milk solution and 3 additional 10 minute washes in PBS/0.5% Tween 20 at room temperature. Antibody detection was performed using Pierce ECL Plus Western Blotting Substrate (Thermo Scientific, 32132).

Antibodies used:

**Primary Antibody**	**Clone no.**	**Cat. no.**	**Source**	**Host**	**Clonality**	**Western Blot dilution**
Anti-Brachyury		AF2085	R&D systems	Goat	Polyclonal	1/500
Anti-Caf1	EPR5576(2)	ab126625	Abcam	Rabbit	Monoclonal	1/1000
Anti-Cdk4	DCS156	2906	Cell Signaling	Mouse	Monoclonal	1/1000
Anti-cleaved caspase-3	5A1E	9664	Cell Signaling	Rabbit	Monoclonal	1/1000
Anti-c-Myc	Y69	ab32072	Abcam	Rabbit	Monoclonal	1/5000
Anti-cyclin A2	BF683	4656	Cell Signaling	Mouse	Monoclonal	1/1000
Anti-cyclin D1	SP4	ab16663	Abcam	Rabbit	Monoclonal	1/200
Anti-cyclin D2	D52F9	3741	Cell Signaling	Rabbit	Monoclonal	1/1000
Anti-cyclin E1	HE12	4129	Cell Signaling	Mouse	Monoclonal	1/1000
Anti-GAPDH	F0911	sc-365062	Santa-Cruz	Mouse	Monoclonal	1/2000
Anti-p21$^{waf1/cip1}$	12D1	2947	Cell Signaling	Rabbit	Monoclonal	1/1000
Anti-p27^{Kip1}	D69C12	3686	Cell Signaling	Rabbit	Monoclonal	1/1000
Anti-Phospho-Rb (Ser807/811)	D20B12	8516	Cell Signaling	Rabbit	Monoclonal	1/1000
Anti-Rb	4H1	9309	Cell Signaling	Mouse	Monoclonal	1/1000
Anti-α-tubulin		T6074	Sigma	Mouse	Monoclonal	1/8000

**Secondary Antibody/ Cat. No.**	**Source**	**Host**	**Species reactivity**	**Clonality**	**Conjugate**	**Western Blot dilution**
ab97051	Abcam	Goat	Rabbit	polyclonal	HRP	1/25000

Real time PCR

Primers used:

QuantiTect Primer Assays<br>(for use in real-time RT-PCR with SYBR Green detection)		
**Gene**	**QuantiTect Primer Assay Name**	**Catalogue number**
beta-actin	Hs_ACTB_1_SG	QT00095431
Brachyury	Hs_T_1_SG	QT00062314
cMyc	Hs_MYC_1_SG	QT00035406
cyclin D2	Hs_CCND2_1_SG	QT00057575
GAPDH	Hs_GAPDH_2_SG	QT01192646
lamin A/C	Hs_LMNA_2_SG	QT01678495
$p21^{Waf1}$/*CDKN1A*	Hs_CDKN1A_1_SG	QT00062090
$p27^{Kip1}$/*CDKN1B*	Hs_CDKN1B_2_SG	QT00998445

CFX Manager™ Version 1.0 software (Bio-Rad) with default parameters was used to assess primer efficiency and specificity and to determine the threshold cycle (Ct) values. Results were normalized to a combination of three endogenous reference genes (GAPDH, β-actin and lamin A/C) and the relative fold change in expression was computed by the ΔΔCt method.

Cell Culture SW480 cells (ECACC, cell line authentication report number 710236782) were cultured in DMEM medium (Life Technologies, #61965) supplemented with 10% fetal calf serum; H460 cells (ECACC, cell line authentication report number 710236782) were grown in RPMI medium (Life Technologies, #61870) supplemented with 10% fetal calf serum. Both cell lines have undergone 16 loci STR authentication (LGC Standards, UK).

Transfection Brachyury siRNA (Qiagen, SI04133521, SI00738255), p27Kip1 (Qiagen, SI02621990), cMYC (Qiagen, SI00300902) and negative control siRNA (Qiagen, 1027280) was used at a final concentration of 5 nM. Transfection was carried out with HiPerFect Reagent (Qiagen, 301705) according to the manufacturer's instructions.

Extreme limiting dilution analysis Extreme limiting dilution analysis (ELDA) was performed as described in (35). Briefly, colonosphere-derived cells were collected from 10 cm dishes and diluted into single-cell suspensions, plated at concentrations of 1000, 100, 10 cells and 1 cell per 100 µl SCM using repeats of

defined experimental conditions in 96-well ultra-low attachment plates (Costar, Corning, 3474). The cells were incubated at 37°C in a 5% CO_2 atmosphere for 10 days. Cells were supplemented with 50 µl of SCM and transfection complexes re-applied after 4 and 8 days of incubation. At the end of 10 days of culture, the number of wells showing formation of colonospheres with more than 20 cells were counted by light microscopy. ELDA webtool (http://bioinf.wehi.edu.au/software/elda) was used to determine frequencies of colonosphere forming cells.

Senescence staining Senescence staining was performed using a Senescence β-galactosidase staining kit (Cell Signaling, #9860) following the manufacturer's protocol.

Immunohistochemistry (IHC) Samples of human colon cancer were obtained from individuals undergoing colonic resection. Written consent was obtained from individual patients and ethical approval was from the local research ethics committee (North Wales Research Ethics Committee – West). The tissue samples were fixed in formalin and embedded in paraffin and IHC analysis was performed on 4 µm tissue sections. Staining was automated on a Ventana Benchmark XT machine using a standard immunohistochemistry protocol. Antigen retrieval and antibody dilutions are listed in Supplementary Table 6. 3,3'-Diaminobenzidine (DAB) was used as a chromogenic substrate, and the slides were counterstained using haematoxylin. Negative controls were omission of the primary antibody. Digital images were captured using the Ventana iCoreo slide scanner (Ventana, Roche)

Primary antibodies for IHC and their dilutions, pre-treatments, and sources:

Antibody	Clone	Host	Dilution	Antigen retrieval/Protocol	Source	Cat. number
Anti-Brachyury	Polyclonal	Rabbit	1:100	Protease I 4 minutes, 1°ab 1hr+Ultra Wash	Abcam	ab20680
Anti-Brachyury	Monoclonal	Mouse	1:750-1:1500	Protease I 4 minutes, 1°ab 1hr+Ultra Wash	Abcam	ab57480
Anti-p27^{Kip1}	Monoclonal	Rabbit	1:2500	STD CC1, 1°ab 1hr +Ultra Wash	Cell Signalling	3686
Anti-ki67	Monoclonal	Mouse	1:300	STD CC1, 1°ab 1hr +Ultra Wash	LSBio	LS-B64433

NOTE. Both CC1 and protease I are Ventana products (Ventana Medical Systems Inc, Tucson, AZ).

RNAseq Total RNA was isolated using RNeasy Plus Mini Kit (Qiagen, #74136) according to the manufacturer's protocol. Total RNA was quality controlled on the Agilent Bioanalsyer RNA 6000 Nano chip and shown to be of good quality (RIN > 9.8). Indexed sequencing libraries were then prepared using the Illumina TruSeq RNA v2 protocol. Briefly, mRNA was enriched on oligo-dT beads before fragmentation and random priming. Reverse transcription was carried out with second strand synthesis and the resultant double stranded cDNA was end repaired, A-tailed and Illumina TruSeq adapters were ligated. Finally, correctly ligated fragments were enriched by performing 12 cycles of PCR with primers complementary to the Illumina adapters. The final libraries were quality controlled and quantified on the Agilent Bioanalyser DNA 1000 chip and Life Technologies Qubit High Sensitivity DNA assay before being pooled to an equimolar concentration of approximately 10 nM. qPCR was performed on the a 105 dilution of the mulitplex pool (Kapa Biosystems Library Quantification Kit) before 12 pM of multiplex library sequenced on one lane of an Illumina HiSeq (TruSeq v3 chemistry) generating 190 million reads passing filter. Reads were demultiplexed and fastq generated using Illumina CASAVA v1.8.2 software.

FACS SW480 cells were transiently transfected with a GFP-encoding SureSilencing shRNA plasmid (Qiagen, KH02753) using the Attractene Transfection Reagent (Qiagen, 301005). The shRNA target sequences were 5'-ACAACTCACCTGCATGTTTAT-3' (vector 1), 5'-TGAGCCTCGAATCCACATAGT-3' (vector 2), 5'-CAAGAACGGCAGGAGGATGTT-3' (vector 3) and 5'-TGCTGAACTCTTGCATAAGT-3' (vector 4) for Brachyury and 5'-GGAATCTCATTCGATGCATAC-3' for negative control vector. Samples were analyzed on a Partec CyFlow® Cube 8 flow cytometry instrument for GFP fluorescence and DNA content. Data was processed using the multicycle cell cycle analysis plug-in for FCS Express 4.
